# Supplementary material for: Crude Extracts of Talaromyces Strains (Ascomycota) Affect Honey Bee (Apis mellifera) Resistance to Chronic Bee Paralysis Virus
Source: Viruses. 2023 Jan 25;15(2):343. doi: 10.3390/v15020343 (PMC9958978; doi:10.3390/v15020343)

Molecular networking analysis

Masses of Rubratoxin A and B were found in clusters but not annotated.

No level 2 annotation can be assigned.

Rubratoxin A

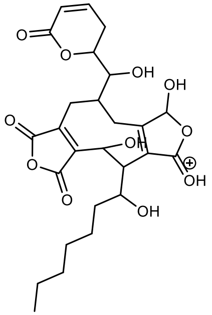

Chemical Formula: $C_{26}H_{33}O_{11}^{+}$

Exact Mass: 521.2017

Rubratoxin B

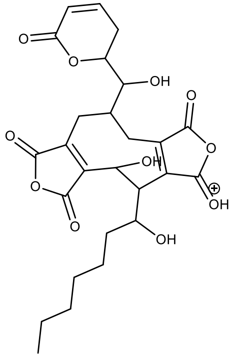

Chemical Formula: $C_{26}H_{31}O_{11}^{+}$

Exact Mass: 519.1861

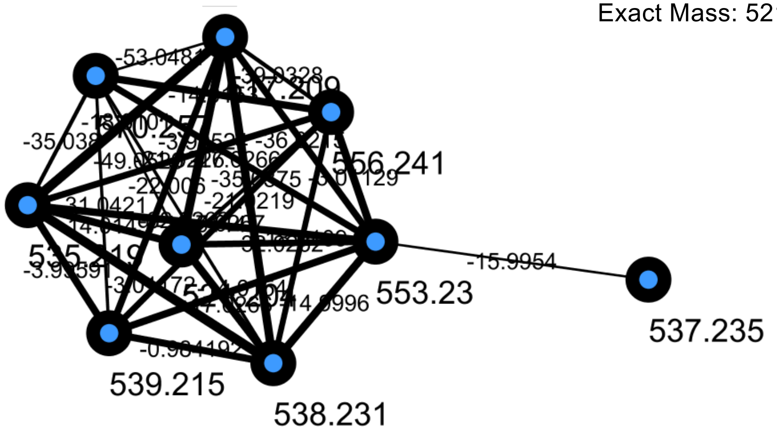

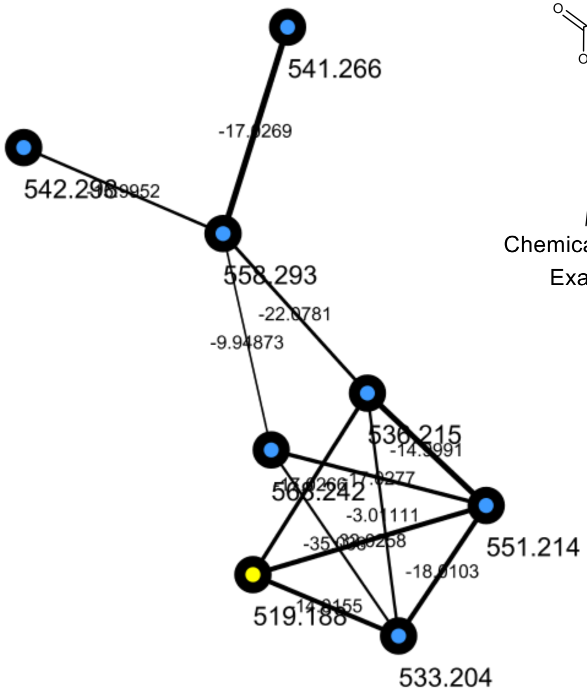

Supplement: Supplementary file 1 [file viruses-15-00343-s001.zip › viruses-2176098-supplementary (2)/Supplementary data_molecular networking.pdf]
